# Urinary Cell-Free miR-99a-5p as a Potential Biomarker for Estrus Detection in Buffalo

**DOI:** 10.3389/fvets.2021.643910

**Published:** 2021-05-17

**Authors:** Aparna Hebbar, Rajeev Chandel, Payal Rani, Suneel Kumar Onteru, Dheer Singh

**Affiliations:** Animal Biochemistry Division, Molecular Endocrinology, Functional Genomics and Systems Biology Laboratory, Indian Council of Agricultural Research -National Dairy Research Institute, Karnal, India

**Keywords:** estrus, buffalo, bta-miR-99a-5p, urine, cell free miRNA, qRT-PCR

## Abstract

Accurate estrus detection method is the need of the hour to improve reproductive efficiency of buffaloes in dairy industry, as the currently available estrus detection methods/tools lack high sensitivity and specificity. Recently, circulating miRNAs have been shown as non-invasive biomarkers by various studies. Hence, in order to evaluate their potential as estrus biomarkers, the objective of this study was to identify and compare the levels of 10 hormone-responsive miRNAs in the urine collected at proestrus (PE), estrus (E), and diestrus (DE) phases of buffaloes (*n* = 3) pertaining to a discovery sample. Among 10 urinary miRNAs, the levels of bta-mir-99a-5p (E/PE 0.5-fold, *P* < 0.05; DE/PE 1.9-fold), bta-miR-125b (E/PE 0.5-fold; DE/PE 0.7-fold), bta-mir-145 (E/PE 1.5-fold; DE/PE 0.7-fold), bta-mir-210 (E/PE 1.2-fold, DE/PE 0.7-fold), mir-21 (E/PE 1.5-fold, DE/PE 2-fold), and bta-mir-191 (E/PE 1.3-fold; DE/PE 0.8-fold) were found to be altered during different phases of buffalo estrous cycle. In contrast, bta-mir-126-3p, bta-let-7f, bta-mir-16b, and bta-mir-378 were undetected in buffalo urine. Furthermore, a validation study in an independent group of 25 buffalo heifers showed the increased levels of urinary bta-mir-99a-5p during the DE (3.92-fold; *P* < 0.0001) phase as compared to the E phase. Receiver operating characteristic curve analyses also revealed the ability of urinary miR-99a-5p in distinguishing the E from the DE phase (area under the curve of 0.6464; *P* < 0.08). *In silico* analysis further showed an enrichment of miR-99a-5p putative targets in various ovarian signaling pathways, including androgen/estrogen/progesterone biosynthesis and apoptosis signaling, implicating the role of miR-99a-5p in ovarian physiology. In conclusion, significantly lower levels of bta-mir-99a-5p at the E phase than the DE phase in buffalo urine indicate its biomarker potential, which needs to be further explored in a large cohort in the future studies.

## Introduction

Dairy farming is one of the most significant parts of the agriculture sector in India. Buffaloes are usually found in Southeast Asia and the Mediterranean region (1). They are preferred over other farm animals in India for their higher financial returns *via* milk and meat, better efficiency in utilizing low-quality feed, and resistance to tropical diseases (1, 2). However, poor reproductive efficiency of buffaloes is one of the major limitations hindering their maximum production potential. Several factors lead to poor reproductive performance of buffaloes, including delayed puberty, silent heat, variation in calving interval, and low conception rate (1, 3), but silent estrus is the major cause of concern (3). In addition, because of lack of an accurate estrus detection method, success of artificial insemination (AI) is limited in buffaloes (4). Generally, estrus is determined by various methods, including hormonal estimation, observation of visual and behavioral signs, gynecoclinical examination, record keeping (5, 6), and various devices (7), but their sensitivity and specificity vary in detecting estrus (8). As per an estimate, ~50% of ovulations remain unnoticed in dairy industry due to diminished estrus behavior (9). Hence, ineffective estrus detection in dairy animals ultimately creates financial loss to the farmers (7). Thus, an accurate estrus detection in buffaloes is essential for effective reproductive management *via* successful conception, which is usually achieved by carrying out AI generally during ovulation that occurs ~10–12 h after the end of an estrus (E) phase in cattle and buffaloes (10). Therefore, there is an urgent requirement to develop a highly sensitive and specific estrus detection method for buffaloes to increase the AI success rate.

MiRNAs are small non-coding RNAs that mainly regulate gene expression at posttranscriptional level. Literature survey showed a cyclic expression of miRNAs in bovine ovarian tissues during an estrous cycle (11, 12), suggesting their specific role in different phases of an estrous cycle. Moreover, miRNAs were also reported to be stably present in extracellular environment, either inside extracellular vesicles such as exosomes or as miRNA protein or miRNA lipoprotein complexes (13–15). Evidences showed the presence of tissue-specific miRNAs in circulation at quantifiable levels (16), indicating their use as a biomarker. For example, higher levels of urinary miR-210-3p in cancer patients were significantly reduced in a disease-free stage (17), demonstrating the tumor as their source of origin. Similarly, decreased plasma levels of miR-222, miR-151-5p, and let-7e after thyroidectomy in papillary thyroid cancer patients indicate their secretion in systemic circulation by tumors (18). In this context, biomarker potential of circulating miRNAs was shown for multiple diseases, including reproductive diseases (19, 20).

In contrast to humans, very few research studies showed the biomarker potential of circulating miRNAs in farm animals. miRNAs have been shown to be involved in reproductive physiology in animals, including regulation of follicular and luteal development (21), pregnancy (22), and follicle-to-luteal transition (23). Moreover, it has been reported that the miRNA profile of the reproductive tissues (11, 23, 24) and plasma of bovine varies in different phases of an estrus cycle (9), which could be due to cyclic variation in ovarian hormones, mainly estrogen and progesterone. As the ovary is highly vascularized organ (9), it is reasonable to hypothesize that miRNAs from ovarian tissue may be excreted differentially and/or specifically in circulation, depending on the phase of an estrus cycle, suggesting that circulating miRNAs may mirror ovarian miRNA profile. In addition, some of these systemic miRNAs may get filtered out *via* the kidney and ultimately appear in urine, and hence, they can be used as estrus biomarkers. Thus, circulating miRNAs in bovine urine may convey the specific phase of an estrous cycle.

Although miRNA profile in bovine plasma (9) and ovarian tissues (23, 25–27) gets altered during an estrous cycle, no study has been conducted until now to identify the altered levels of urinary miRNAs during bovine estrous cycle. Therefore, the present study was planned to explore the altered levels of urinary miRNA during estrous cycle considering the buffalo as a model. In this study, we used quantitative reverse transcriptase–polymerase chain reaction (qRT-PCR) to detect the levels of 10 hormone-responsive miRNAs, which were selected on the basis of their implication in ovarian physiology as per the available scientific literature, i.e., miR-125b (28), miR-99a (29), miR-145 (30), miR-21 (31), miR-191 (30), miR-210 (32), let-7f (30), miR-16 (33), miR-378 (30), and miR-126 (34) in buffalo urine and compared their levels across different phases of an estrous cycle. In addition, validation of urinary miR-99a-5p levels was performed in a separate group of 25 buffaloes. At last, *in silico* analysis was done to identify putative targets of miR-99a-5p using miRwalk 2.0 followed by their association analysis with different cellular signaling pathways using Panther, an online tool.

## Materials and Methods

### Experimental Animals

For the discovery phase of the study, three buffalo heifers were managed as per the standard conditions at the Livestock Research Centre, ICAR–National Dairy Research Institute (NDRI), Karnal. In addition, 25 animals, which were presented for AI at the AI center, ICAR-NDRI, were considered for the validation study. The study was approved by the NDRI Institutional Animal Ethics Committee (approval no. 42-IAEC-18-2).

#### Sample Collection and Processing

Urine and saliva samples were collected in the morning time and in the evening on the day of E (day 0), proestrus (PE) (day −2), and diestrus (DE) (day 10). In case animals did not urinate for 20 min, the animals were stimulated for urination by pouring water on the rump region or by tying the animal or by offering the water to the animal. In brief, midstream urine was collected from healthy buffalo heifers (*n* = 3 for discovery group and *n* = 25 for validation group) in 50 mL centrifuge tubes, transported to the laboratory on ice, and centrifuged at 3,000 *g* for 5 min at 4°C. The supernatant or cell-free urine (CFU) was transferred to another microcentrifuge tube and then either used immediately or stored in −20°C until further use. In case of the discovery group, urine was collected from different phases of three consecutive estrous cycles of each animal, but the samples that belonged to the middle estrous cycle were selected for further analysis. In validation group, urine was collected from buffaloes on day 0 before insemination and again on day 10. Estrus and DE paired samples from animals that showed typical salivary fern pattern of estrus on day 0 and were found to be non-pregnant during 60 days after AI were included in this study.

### Detection of an Estrous Cycle Phase

Buffaloes were observed for estrus symptoms twice a day in the morning and the evening for the period of 6 months. E phase was determined by observing various signs such as visual observation (vaginal discharge, hyper salivation, and abnormal posture), gynecoclinical examination (cervical relaxation and uterine tonicity), and biochemical confirmation (cervical mucus crystallization or salivary fern pattern) and rated as either mild, moderate, or intense as mentioned in **Table 2**. Serum (*n* = 3) was used to estimate the estradiol levels using enzyme-linked immunosorbent assay–based Estradiol Estimation Assay kit (ADI-900-174 by ENZO) as per the manufacturer's protocol.

### Saliva Collection and Fern Pattern Analysis

**Figure 1:**
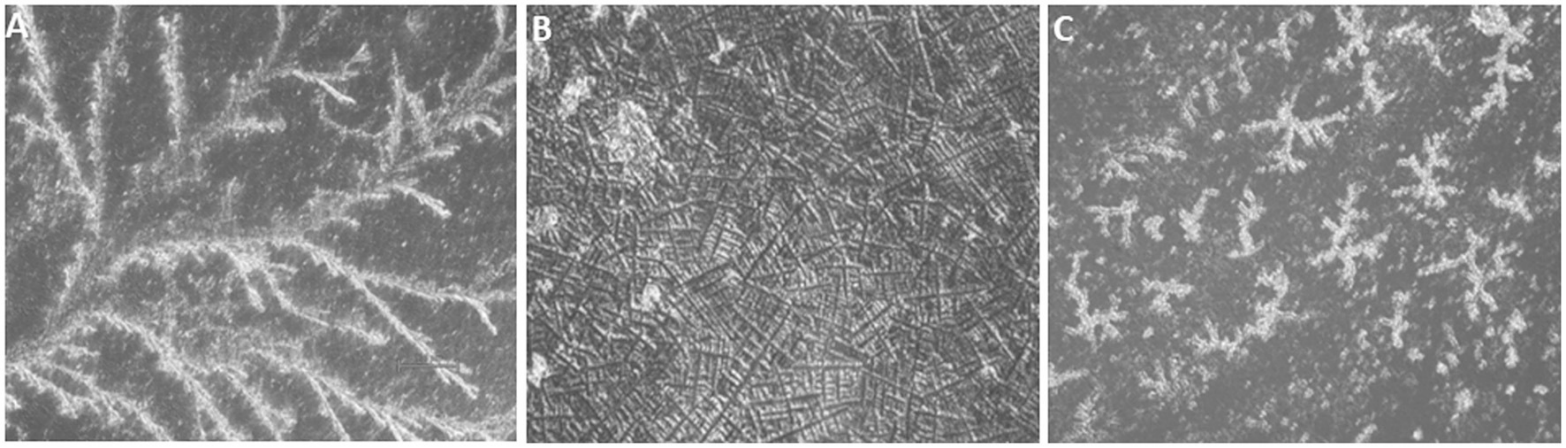
Typical salivary crystallization of buffalo (air dried, ×200). **(A)** Salivary crystallization during the proestrus phase. **(B)** Typical fern patterns of saliva during the estrus phase. **(C)** Discontinuous and improper crystallization during the diestrus phase.

Unstimulated saliva was collected from the lower lip of buffaloes before feeding time in the morning on every alternative day of the estrous cycle, brought to the laboratory on ice and centrifuged at 3,000 *g* for 5 min at 4°C. A 10 μL of cell-free saliva was used for smear preparation on a clean glass slide for observing the salivary fern patterns under an inverted microscope (Nikon Eclipse Ti-S, Japan) to determine the phase of an estrous cycle as per our previous study (2) (Figure 1).

### Total RNA Isolation

RNA was extracted from CFU using TRIzol LS (Life Technologies, USA) as per the manufacturer's protocol. In brief, 250 μL of CFU was mixed with 750 μL of TRIzol LS and vortexed briefly, and the mixture was kept at room temperature for 5 min. Then, 200 μL of chloroform (Sigma Chemicals Co., catalog no. C2432) was added to the mixture, vortexed for 15 s, and incubated for 15 min at room temperature followed by a centrifugation at 12,000 relative centrifugal force (rcf) for 15 min. Aqueous supernatant was transferred to a fresh Eppendorf tube, 500 μL of isopropanol was added to it, and the mixture was incubated at −20°C for 30 min. Later, it was centrifuged at 12,000 rcf for 10 min. The supernatant was discarded, and the pellet was washed with 70% ethanol. Finally, the RNA pellet was dissolved in 20 μL of nuclease-free water and then either used immediately or stored at −20°C until further use. RNA purity and concentrations were determined by using a Nanodrop spectrophotometer.

### cDNA Synthesis

Total RNA was used to prepare cDNA as per the miScript II RT kit (catalog no. 218161, Germany) according to the manufacturer's protocol. In brief, 10 μL of cDNA reaction mixture containing 500 ng of the total RNA, 2 μL of 5X miScript Hispec buffer, 1 μL of RT mix, 1 μL of 10 × miScript nucleics mix provided by Qiagen miscript II kit (catalog no. 218161, Germany), and 1 μL of an exogenous spiked-in miRNA control, syn-cel-miR-39-3p (1 μL; miRNeasy Serum/Plasma Spike-In Control, catalog no. 219610, Qiagen Co., Germany) was incubated at 37°C for 60 min and 95°C for 5 min. The prepared cDNA was kept at −20°C until further use.

### Real-Time PCR

Urinary levels of miRNAs were determined by qRT-PCR with some modifications using either Light Cycler 480 II (Roche, CA, USA) or Applied Biosystems Fast 7500 Real-Time PCR system (Roche, CA, USA). The primers used for miRNA amplification were designed on the basis of mature miRNA sequences of bovine using miRBase 21 and procured from a commercial firm (Table 1) (35). In brief, 12 μL PCR reaction mixture consisting of 5 μL of cDNA (1:20 diluted), 5 μL of 2 × Quantilect SYBR Green PCR Master Mix, 1 μL of 5 μM miRNA-specific primer, and 1 μL of 10 × miScript Universal Primer (catalog no. 218073, Germany) was incubated at 95°C for 15 min, followed by 40 cycles of 94°C for 15 s, 55°C for 30 s, and 70°C for 30 s. The melt curve analysis was performed at the temperature ranging from 70 to 95°C. Each sample was run in duplicate. qRT-PCR data analysis was done using 2^−Δ*ΔCt*^ method (36) by using cel-miR-39 as an exogeneous spiked-in miRNA for normalization (13).

**Table 1:** miRNAs primers used in the study.

**Gene name**	**Primer name: sequence (5^**′**^ to 3^**′**^)**	**Accession no**.
bta-miR-99a-5p	AACCCGTAGATCCGTTCTTGT	MIMAT0003537
bta-mir-145	GTCCAGTTTTCCCAGGAATCCC	MIMAT0003542
bta-mir-125b	TCCCTGAGACCCTAACTTGTGA	MIMAT0003539
bta-mir-126-3p	CGTACCGTGAGTAATAATGCG	MIMAT0003540
bta-miR-21-5p	GCTTATCAGACTGATGTTGAC	MIMAT0003528
bta-let-7f	TGAGGTAGTAGATTGTATAGTT	MIMAT0003519
bta-mir-210	ACTGTGCGTGTGACAGC	MIMAT0003824
bta-mir-378	ACTTGGAGTCAGAAGGC	MIMAT0009305
bta-miR-191	CAACGGAATCCCAAAAG	MIMAT0003819
bta-mir-16b	TAGCAGCACGTAAATATTGG	MIMAT0003525

#### Target Gene Prediction and Pathway Analysis for miR-99a-5p

MiRWalk, an online tool, was used to predict putative mRNA targets that might be regulated by miR-99a-5p under a threshold of 3 (37). The resultant predicted targets for miR-99a-5p were run through Protein Analysis Through Evolutionary Relationship (PANTHER) classification system and analysis tools (38) in order to identify their associated biological processes considering the bovine as a genome background.

### Statistical Analysis

Statistical analyses were performed either by one-way analysis of variance (ANOVA) followed by *post-hoc* Tukey test or paired *t*-test using GraphPad Prism software 5.1. (GraphPad Software, Inc., San Diego, CA, USA). Results are shown as the mean ± SEM. Receiver operating characteristic (ROC) curve analysis was performed using ΔCt values of the miR-99a-5p in the E and DE phase samples.

## Results

### Intensity of Estrus Signs

Estrus was determined by various physical indicators such as tonicity of uterus, swelling of vulva, vaginal discharge, and typical salivary fern patterns. Physical indicators were categorized as mild, moderate, and intense as shown in Table 2. Among three buffaloes at the E phase, moderate or intense swelling of vulva was recorded in 66.67% of cases together. Similarly, estrus intensity indicators such as vaginal discharge and salivary ferns were observed to be either moderate or intense in all animals (100%). Tonicity of uterus was found to be moderate in 100% of the animals. Serum estradiol levels were further determined at E phase in buffaloes (Table 3), which were found to be significantly higher at the PE (29.58 ± 2.591 pg/mL) and E phases (21.39 ± 2.624 pg/mL; *P* < 0.05) as compared to the DE phase (16.28 ± 1.174 pg/mL).

**Table 2:** Intensities of overt estrous signs in different buffaloes.

**Animal no**.	**A1**	**A2**	**A3**
Swollen vulva	+	++	++
Vaginal discharge	++	++	+++
Salivary fern	++	++	+++
Tonicity of uterus	++	++	++

*Estrus intensities were determined by visual behaviors and biochemical and gynecoclinical parameter as mild (+), moderate (++), and intense (+++)*.

**Table 3:** Estradiol levels during different phases of an estrous cycle in buffaloes.

**Animal no**.	**A1**	**A2**	**A3**	**Average**	**SE**
PE	26.9	27.08	34.76	29.58	2.591
E	21.35	16.87	25.96	21.39	2.624
DE	14.39	16.01	18.43	16.28	1.174

### miRNA Urinary Levels

**Figure 2:**
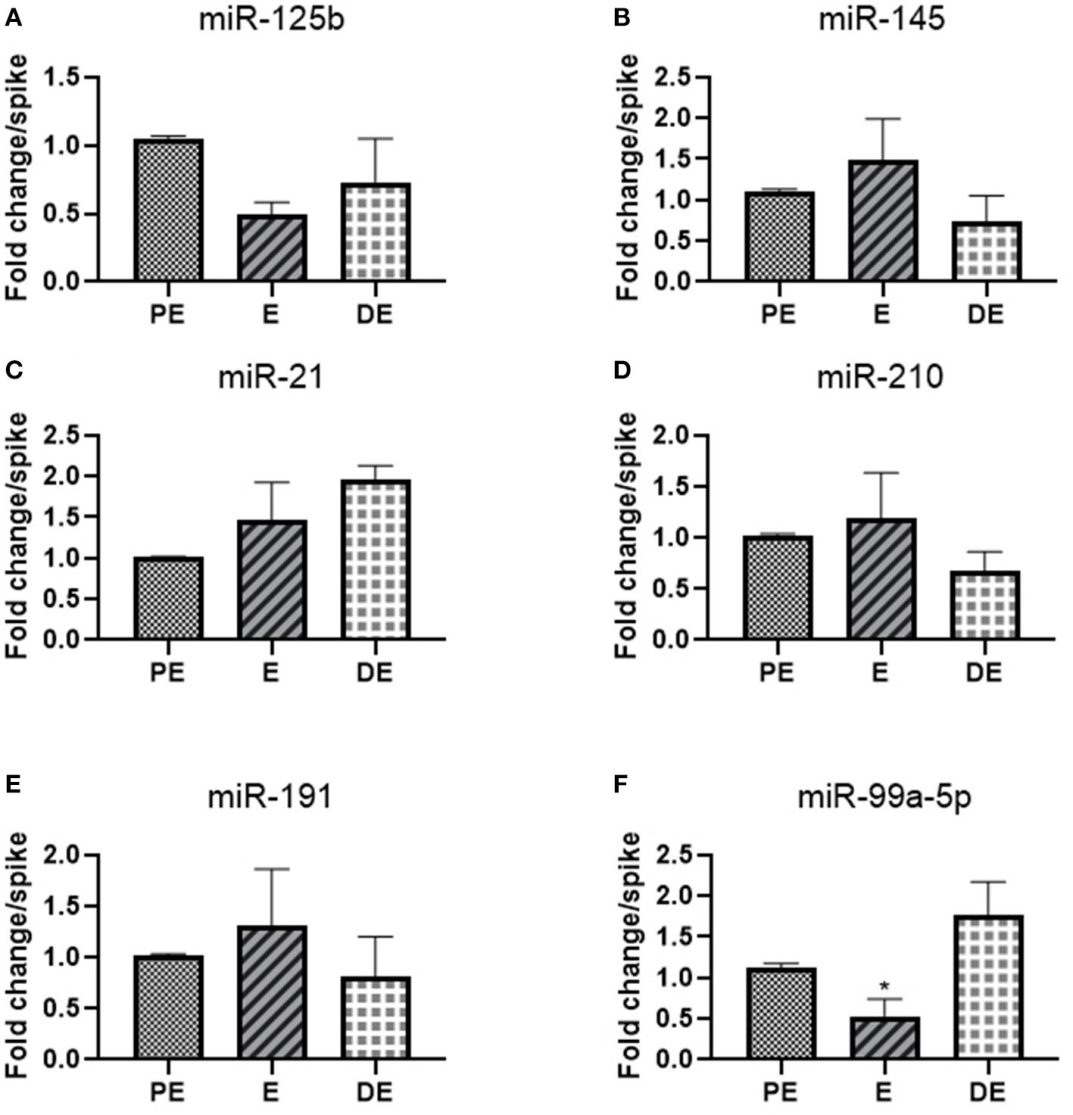
Real-time PCR analysis of miRNAs **(A)** miR-125b, **(B)** miR-145, **(C)** mir-21, **(D)** mir-210, **(E)** miR-191, and **(F)** miR-99a-5p in proestrus (PE), estrus (E), and diestrus (DE) phases. One-way ANOVA test was done followed by *post-hoc* Tukey test for analysis. Each bar in the figure represents the mean ± SE of three independent experiments *P < 0.05.

We selected 10 hormone-responsive miRNA candidates identified on the basis of literature (Table 2) for qRT-PCR analysis. Unfortunately, the urinary levels of let-7f, mir-126-3p, miR-378, and mir-16b were too low to be detected by qRT-PCR. Of the remaining six miRNAs, mir-145 (1.5-fold E/PE; 0.7-fold DE/PE), mir-21(1.5-fold E/PE; 2-fold DE/PE), miR-210 (1.2-fold E/PE; 0.7-fold DE/PE), mir-191 (1.3-fold E/PE; 0.8-fold DE/PE), and miR-125b (0.5-fold E/PE; 0.7-fold DE/PE) did not significantly change during the E cycle (Figure 2). In contrast, miR-99a-5p levels increased at the DE phase (1.9-fold) and decreased at the E phase (0.5-fold *P* < 0.05) compared to the PE phase as shown in Figure 2.

### Validation of mir-99a-5p Urinary Levels and ROC Curve Analysis

**Figure 3:**
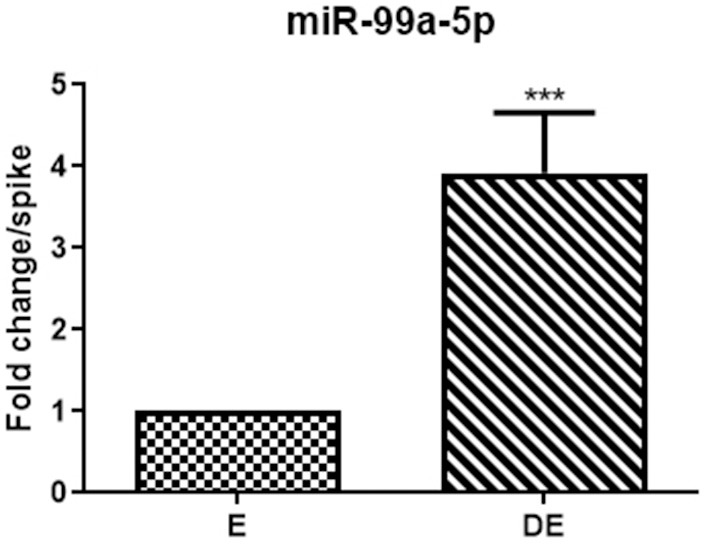
Real-time PCR analysis of mir-99a-5p in the validation population of the animals in estrus (E) and diestrus (DE). The paired *t*-test was used for analysis. Each bar represents the mean ± SEM of 25 independent experiments. ***P < 0.001.

**Figure 4:**
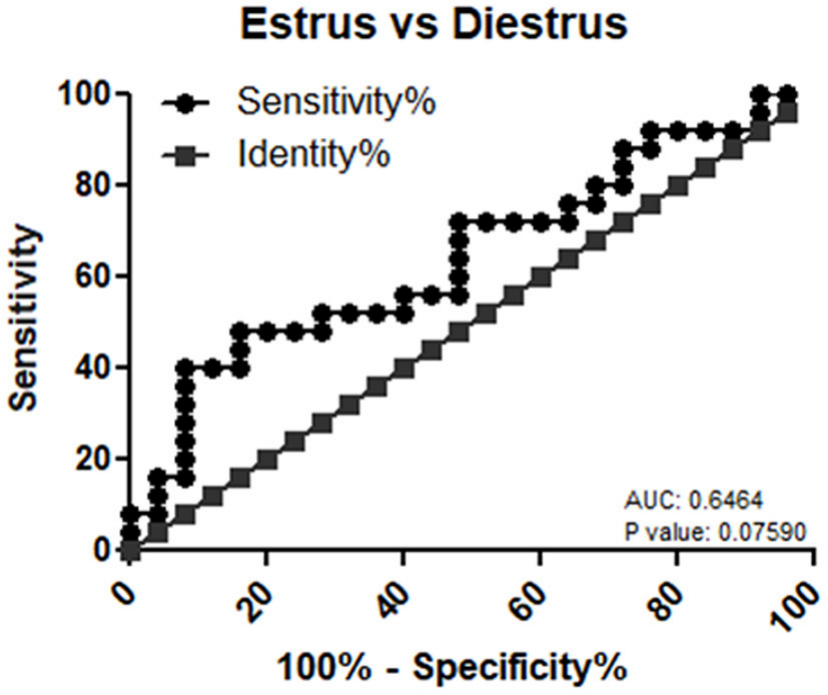
Receiver operating characteristic (ROC) curve of miR-99a-5p as a diagnostic marker for estrus.

mir-99a-5p levels were analyzed in another group of 25 buffaloes on the day of the E and DE phases during an estrous cycle. mir-99a-5p levels were observed to be significantly increased (3.92-fold; *P* < 0.0001) in the DE phase as compared to the E phase as shown in Figure 3. Finally, we determined discriminatory performance of miR-99a-5p using ROC curve analysis as shown in Figure 4. E and DE datasets were used to make ROC curve. ROC curve analysis showed that at the 7.962 cutoff value, the sensitivity and specificity of miR-99a-5p in differentiating the E from DE phase were 56 and 60%, respectively; the AUC was 0.6464 (95% confidence interval, 0.4921–0.8007; *P* = 0.076) as shown in Figure 4. ROC curve results suggest the potential of miR-99a-5p to distinguish the E and DE phases.

#### Target Gene Prediction and Pathway Analysis of miR-99a-5p

**Figure 5:**
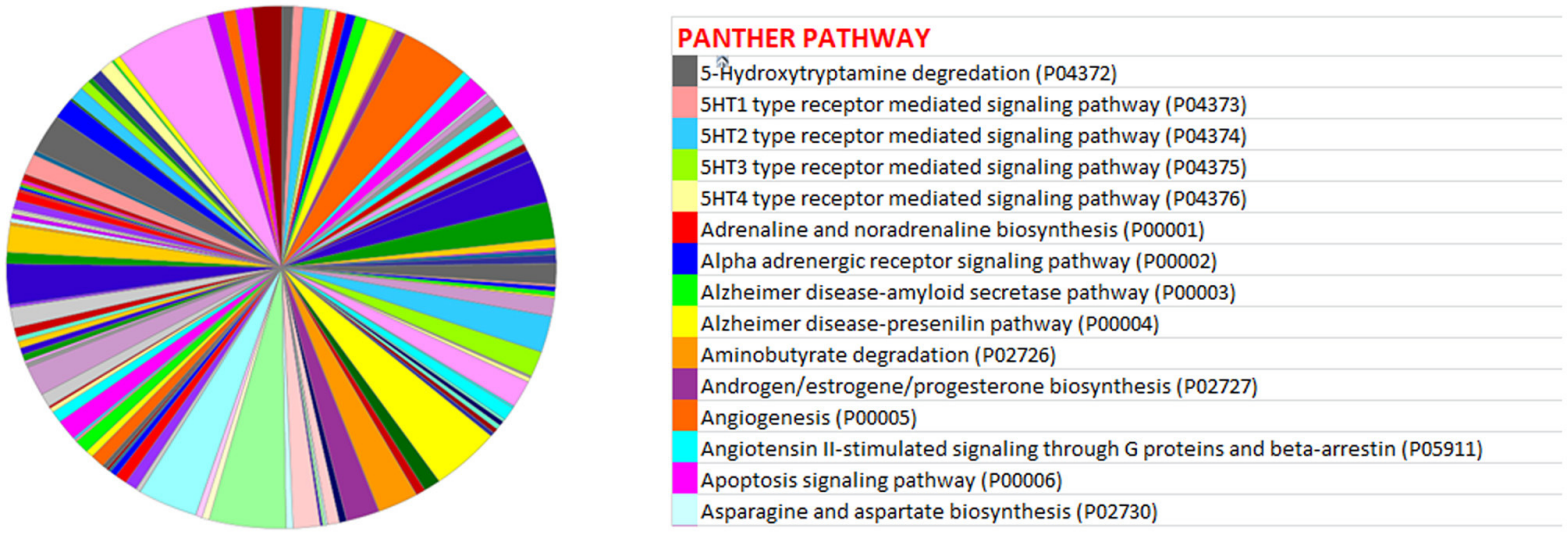
Association of bta-miR-99a-5p target genes with different pathways as shown in the pie chart by PANTHER, an online tool.

**Figure 6:**
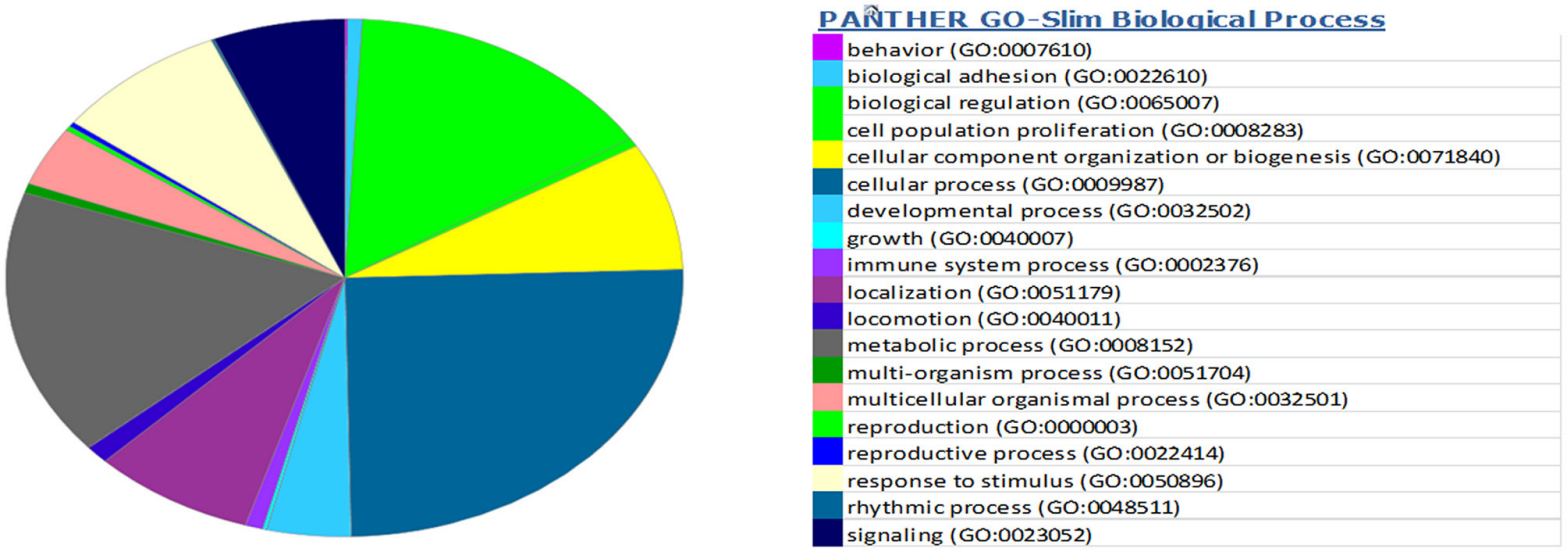
Association of bta-miR-99a-5p target genes with different biological processes as shown in the pie chart by PANTHER, an online tool.

miRwalk, an online tool, predicted 7,347 target genes for miR-99a-5p (Supplementary Table 1). miR-99a-5p target sites were identified in the 5′ UTR, CDs, and 3′ UTR regions of putative targets. Among 7,347 predicted target genes, 3,086 putative target genes having NM_Accession prefixes (Supplementary Table 2) were shown to be associated with various cellular pathways (Supplementary Table 3) and biological processes (Supplementary Table 4) by the PANTHER software. Among the top 15 cellular pathways predicted to be regulated by miR-99a-5p, androgen/estrogen/progesterone biosynthesis and apoptosis signaling pathways have been previously implicated in ovarian physiology (Figure 5). Similarly, biological process annotations by PANTHER analysis showed that miR-99a-5p may regulate reproduction and reproductive process (Figure 6).

## Discussion

Currently, an accurate estrus detection biomarker is the need of the hour for effective reproductive management of buffaloes. Recently, a number of studies reported biomarker potential of circulating miRNAs in biofluids owing to their high stability (39) and good clinical performance (40). Among the various miRNAs evaluated in the present study, we found low levels of miR-99a-5p and bta-mir-125b on the estrus day as compared to the PE and DE phases. In contrast, earlier studies showed a higher expression of miR-99a-5p and bta-mir-125b in ovaries during follicular phase and their higher plasma levels during the E phase in cows (9). Another study reported an upregulation of miR-125b expression by androgens, thereby suppressing the follicular atresia *via* targeting a proapoptotic gene (28). Moreover, decreased expression of mir-125b was found in granulosa cells of primordial follicles as compared to primary, secondary, and antral follicles in mouse (41).

In addition, we also found high levels of mir-145, mir-210, and mir-191 on the estrus day as compared to the PE phase. In concordance with our data, higher expression of mir-145 (23, 25) and mir-210 (32) were shown in ovaries during follicular phase, and higher plasma levels of mir-145 were reported during the E phase of cows (9). Furthermore, our study showed a gradual increase of miR-21 levels from the PE to the E phase, followed by its highest levels at the DE phase, which is not in agreement with earlier studies as miR-21 expression in the granulosa cells was found to be lowest on day 14 and at higher levels on day 3 of an estrous cycle (42). Study done by Donadeu et al. (43) reported higher expression of miR-21-5p/-3p in atretic than healthy follicles of cattle. In this context, studies reported the regulation of follicular atresia by miR-21-3p through targeting FGF2 (44) and VEGFA (45) that ultimately inhibits bovine granulosa cell autophagy by repressing AKT/mTOR signaling and PI3K/AKT signaling, respectively. Another study reported the higher expression of bta-miR-21-5p during early CL (1–7 days) as compared to middle CL and late CL (46), suggesting its role in the corpus luteum formation, functionalization, and corpus luteum regression (23). Luteinizing hormone was reported to upregulate antiapoptotic miR-21 in murine granulosa cells, suggesting its role in granulosa cell survival and differentiation (47). Moreover, elevated levels of miR-21, miR-145, and miR-378 in follicular fluid aspirated from dominant follicles during seasonal anovulation as compared to the seasonal ovulation period in mares further suggest their role in follicle maturation (48). Literature survey also showed the higher levels of miR-125b (23, 27) and miR-145 (23, 25) and low levels of miR-21 (23, 26) in follicular tissues as compared to luteal tissues in the ovaries, indicating their role in follicle-to-luteal transition. At last, let-7f, miR-16b, miR-126-3p, and mir-378 were not detected in buffalo urine, suggesting their levels might be below the detection limit of qRT-PCR or completely absent. In this context, Weber et al. (49) reported that urine generally has the lowest amount of miRNAs among 12 different biological fluids.

Hormone plays an important role in estrous cycle regulation. In this context, multiple studies showed a reciprocal relationship between miRNAs expression/levels and hormones. One of the studies suggested partial regulation of circulating miR-125b, miR-99a, miR-145, and miR-378 by hormones in hyperstimulated heifers (50). Hu et al. (51) reported downregulation of miR-125b expression in response to 17α-E2. Reciprocally, miRNAs also regulate ovarian hormone biosynthesis and release. Sirotkin et al. (52) demonstrated the role of miRNAs in the regulation of estradiol (miR-125b and miR-126), testosterone (miR-16, miR-145, miR-125b, miR-21, and miR-126), and progesterone (miR-16, miR-145, miR-125b, and miR-126) release, respectively, in granulosa cells. Zhang et al. (53) reported that decreased expression of miR-125b-5p stimulates testosterone secretion and decreases the estradiol release in mouse preantral follicles *via* regulation of PAK3/ERK1/2 signaling. Hence, it can be concluded that miRNAs and hormonal interplay might be involved in estrous cycle regulation. Moreover, multiple investigations showed the extracellular presence of the miRNAs evaluated in the present study. Naji et al. (54) reported that higher levels of miR-145 in follicular fluid can be used as a predictive biomarker for polycystic ovary syndrome. Singh et al. (55) reported the lower levels of salivary mir-16b in the presence of dominant ovarian follicle. Li et al. (34) reported higher serum levels of miR-126-3p during ovulation and midluteal phase in comparison to early follicular phase. A study conducted with cow plasma demonstrated the significantly increased levels of miR-99a-5p, let-7f, miR-145, and miR-125b in estrus as compared to the other phases during estrous cycle of cows (9). Taken together, the miRNA presence in biofluids further suggests their potential use as a biomarker.

Among the studied miRNAs, miR-99a-5p can be used as an estrus biomarker. Tripurani et al. (56) reported that the higher expression of miR-99a in ovarian tissue suggested its role in basic reproductive activities. Geng et al. (57) reported decreased proliferation and increased apoptosis of granulosa cells by miR-99a *via* targeting insulin like growth factor 1R, suggesting its expression might be low during folliculogenesis, which could partly explain its low urinary levels during estrus in buffaloes. Literature survey also indicated the role of miR-99a in cell cycle (58) and glucose metabolism (59). Noferesti et al. (50) reported hormonal regulation of circulating miR-99a, which further explains its dynamic urinary levels during an estrous cycle due to hormonal cyclicity. The functions of miR-99a-5p have not been reported completely as yet in relation to fertility and physiological functions in the ovary; we herein speculate its possible role by predicting the association of miR-99a-5p targets with different cellular pathways, including those pathways associated with biosynthesis of androgen, estrogen, and progesterone hormones and apoptosis, which ultimately suggests its role in the reproductive process. Hence, *in silico* analysis gives clues regarding the implication of miR-99a-5p in ovarian physiology. Moreover, ROC curve analysis gave a solid support that miR-99a-5p could clearly differentiate between the E and DE phase. At last, its dynamic presence in plasma and urine during the estrous cycle as shown previously further hints toward its definite potential as an estrus biomarker in the near future. Thus, these data explained why miR-99a could serve as a promising biomarker for estrus detection in buffaloes.

In general context, lack of agreement between the present and numerous previous studies might be due to difference in preanalytical and analytical variables used, including sample source, RNA isolation protocols, RNA quality and quantity, cDNA synthesis kits, miRNA quantification techniques, and normalization gene (60, 61). For example, miRNA profile varies according to different body fluids. For example, an miRNA profile in urine and plasma samples from the same animal may vary, as urine being the ultrafiltrate of plasma. In addition, we stored CFU in −20°C, which may affect RNA yield, as urinary Extracellular Vesicles (EVs) or naked miRNAs may get entrapped inside Tamm–Horsfall protein complex present in the urine under low temperature conditions and subsequently get lost during RNA isolation (62). Currently, there is no consensus regarding ideal reference gene that should be used for qRT-PCR data normalization in case of biofluids. In the present study, exogenous cel-mir-39 was used for qRT-PCR data normalization, which may reduce variability caused by differences in reverse transcription efficiency across samples, but it is insufficient to remove technological variability across samples caused by various factors including collection, handling, and storage of samples. At last, cell-free miRNA profile and their levels in urine are dynamic in nature and depend on various factors, including intervariability and intravariability among animals, time point of urine collection, and seasonal variation. Hence, there is always a chance of getting variable miRNA profile from urine taken at the same phase of an estrous cycle.

Our study has a number of limitations. First, our study is not comprehensive in terms of cell-free miRNAs profiling present in buffalo urine, although it showed the biomarker potential of urinary miR-99a-5p for estrus detection. Second, we used a small number of samples during the discovery and validation phase; hence, our finding needs to be verified in a large sample size. Third, our results must be verified in different breeds of buffaloes. Fourth, urinary levels of miR-99a-5p were found to be differentially altered at different phases of estrous cycle in buffalo, but the origin of urinary miR-99a-5p needs to be elucidated in order to draw a significant conclusion regarding its biomarker potential, as it is already reported to be expressed in numerous other tissues (56) apart from the ovary. Hence, the relative contribution of these organs to urinary miR-99a-5p is not known. Similarly, cell-specific expression of miR-99a-5p in ovarian tissue must be determined in order to better estimate its potential as an estrus biomarker. Fifth, it needs to be deciphered how miR-99a-5p enters the circulation, especially the urine.

In the future, successful clinical use of urinary cell-free miRNAs requires an establishment of guidelines and protocols for urine sampling, processing, storage, and transport; miRNA isolation, normalization, and quantification methods; and miRNA profiling using high-throughput detection techniques. Meanwhile, urinary miRNA diversity and their levels in different phases of an estrous cycle need to be determined for the development of estrus detection biomarker in buffaloes. At last, the biomarker potential of urinary cell-free mir-99a-5p needs to be deciphered in combination with other miRNA panels to increase its sensitivity and specificity for heat detection in the buffalo.

In summary, our study yielded (i) a first insight of hormone-responsive miRNAs levels in buffalo urine using real-time PCR, (ii) a potential of urinary cell-free miR-99a-5p as estrus biomarker in bovine, and (iii) a clue that ovarian-derived miRNAs may get filtered out in buffalo urine. Most importantly, ROC curve analysis showed that urinary levels of miR-99a-5p in buffalo can moderately distinguish the E from DE phases of an estrous cycle, further suggesting its biomarker potential in estrus detection that needs to be further explored in future either alone or in combination with other miRNAs to improve its sensitivity and specificity.

## Conclusion

The present study reported significantly lower levels of urinary cell-free mir-99a-5p at the E phase as compared to the DE phase in buffalo. This may be helpful for the development of mir-99a-5p–based estrus detection kit in the near future.

## Data Availability Statement

The datasets presented in this study can be found in online repositories. The names of the repository/repositories and accession number(s) can be found in the article/Supplementary Material.

## Ethics Statement

The animal study was reviewed and approved by IAEC, NDRI, Karnal.

## Author Contributions

AH and PR: performed the research, methodology, and data analysis. RC: writing—original draft. SO: conceptualization and supervision. DS: conceptualization, supervision, and experimental design. All authors contributed to the article and approved the submitted version.

## Conflict of Interest

The authors declare that the research was conducted in the absence of any commercial or financial relationships that could be construed as a potential conflict of interest.
